# Knowledge and Confidence of a Convenience Sample of Australasian Emergency Doctors in Managing Dental Emergencies: Results of a Survey

**DOI:** 10.1155/2015/148384

**Published:** 2015-03-04

**Authors:** Hossein Samaei, Tracey Joy Weiland, Stuart Dilley, George Alexander Jelinek

**Affiliations:** ^1^Department of Emergency Medicine, St. Vincent's Hospital, Melbourne, VIC 3065, Australia; ^2^Emergency Practice Innovation Centre, St. Vincent's Hospital, Melbourne, VIC 3065, Australia; ^3^Department of Medicine, Dentistry and Health Sciences, The University of Melbourne, Melbourne, VIC 3010, Australia

## Abstract

*Background*. We aimed to determine Australasian Specialist Emergency Physicians' and Emergency Physicians in Training (Trainees') level of knowledge of common dental emergencies. We also explored confidence in managing dental emergencies; predictors of confidence and knowledge; and preferences for further dental education.* Methods*. A questionnaire was distributed electronically (September 2011) and directly (November 2011) to Fellows and Trainees of the Australasian College for Emergency Medicine. It explored demographics, confidence, knowledge of dental emergencies, and educational preferences.* Results*. Response rate was 13.6% (464/3405) and college members were proportionally represented by region. Fewer than half (186/446; 42%) had received dental training. Sixty-two percent (244/391, 95% CI 57.5–67.1) passed (>50%) a knowledge test. More than 60% incorrectly answered questions on dental fracture, periodontal abscess, tooth eruption dates, and ulcerative gingivitis. Forty percent (166/416) incorrectly answered a question about Ludwig's Angina. Eighty-three percent (360/433) were confident in the pharmacological management of toothache but only 26% (112/434) confident in recognizing periodontal disease. Knowledge was correlated with confidence (*r* = 0.488). Interactive workshops were preferred by most (386/415, 93%).* Conclusions*. The knowledge and confidence of Australasian Emergency Physicians and Trainees in managing dental emergencies are varied, yet correlated. Interactive training sessions in dental emergencies are warranted.

## 1. Introduction

Patients presenting to the emergency department (ED) with dental problems account for 0.3 to 4% of ED visits [1–4]. In one United Kingdom (UK) study [5], dental problems were the second most common reason (after drug reactions) patients telephoned the ED for advice. The most common dental conditions presenting to ED were dental pain, dental infections, dental and maxillofacial trauma, and postdental treatment-related complications such as haemorrhage and dry socket [2, 6–8].

Emergency department medical staff may have difficulty making a specific diagnosis for dental presentations, and often manage these patients symptomatically [9]. This may be because they have had little training in dental emergencies and little exposure to these conditions. A survey of newly graduated physicians working in hospitals in Bahrain, Ireland, Kuwait, and the UK reported that 97% had not received any dental education prior to graduation [10]. In a large study from the United States involving 1030 pediatricians and family physicians, most respondents had less than two hours of preventive dental education during their whole medical training [11]. A further study from England showed that only 6% of ED medical staff had received any dental training in medical school [12].

In a cross sectional, nationwide survey that included 118 doctors working at Welsh emergency departments, knowledge for the management dental trauma was found to be partial, with greater knowledge associated with experience and training [13].

In a small US study of 72 physicians and directors, knowledge was poor for dental fractures, and good for luxations and avulsions. Specialist qualification in paediatric emergencies was found to be associated with great knowledge in managing dental trauma [14].

Some studies suggest that emergency physicians with inadequate training and insufficient knowledge had difficulty with diagnosis, investigation, management, and appropriate referral of dental conditions [1, 15].

An assessment of Australasian ED staff knowledge and confidence is lacking and to our knowledge there are no known studies of factors that predict knowledge and confidence in dental emergencies. An understanding of these factors may permit the targeting of relevant educational resources. In addition, awareness of emergency doctors' views about preferred activities which can improve these weaknesses may enable decision makers to consider suitable teaching methods to improve knowledge and confidence.

We aimed to determine specialist emergency physicians' (fellows') and emergency physicians in training (trainees') knowledge of common dental emergencies. Secondary aims were to assess respondent's self-rated confidence in managing dental emergencies, to determine predictors of knowledge and confidence in managing dental emergencies, to determine the degree of correlation between knowledge and confidence and to determine attitudes towards further education in dental emergencies.

## 2. Methods

### 2.1. Study Design and Ethics

This cross sectional survey was approved by St. Vincent's Hospital Melbourne's Ethics Committee and by the Scientific Committee of the Australasian College for Emergency Medicine (ACEM).

### 2.2. Inclusion and Exclusion Criteria

Participants eligible for inclusion in the study were doctors registered with ACEM including Emergency Physicians (“fellows” or “FACEM”), advanced trainees, and provisional trainees. Doctors not registered with ACEM were excluded.

### 2.3. Tool Development and Validation

A questionnaire was designed based on dentistry exam multiple choice questions [16–18], emergency medicine textbooks [19–21], and internet teaching sites. Face validity was ensured using iterative feedback from six ED consultants, two dentists, a researcher, and an emergency medicine trainee after which minor changes were made to the original survey. To ensure content validity, a sample of six emergency physicians and five trainees rated relevance of survey items on a four-point scale. This enabled the identification of items not to be centrally relevant to the topic, items requiring revision in formatting or wording, and items requiring no alteration.

The final questionnaire (see the appendix) comprised 40 items including nine demographic items, 11 items about confidence in diagnosis and management of some of the most frequent dental presentations, 15 multiple choice questions assessing participants' knowledge of common dental emergencies, and five questions regarding desired further training in dental emergencies. The survey incorporated graded responses using Likert scales for attitudinal questions, binary response formats for demographics, and multicategory format (demographics, knowledge questions, and further training in dental emergencies). Some open-ended questions were also included.

### 2.4. Survey Distribution

The electronic survey, formatted using online SurveyMonkey software, was distributed in September, 2011, by email by the ACEM. Electronic surveys were delivered via hyperlink to the online questionnaire embedded in the email. Due to the rules of the ACEM who distributed the survey on behalf of the authors, only one reminder was sent (October 2011). For ethical and privacy reasons the authors were restricted from making direct contact with potential respondents via electronic means. For these reasons, the authors were limited in maximizing the response rate. The electronic survey remained open until January, 2012.

Although not permitted to send more than one reminder, the authors were able to disseminate a paper based version of the survey to conference registrants at the ACEM Annual Scientific Meeting in Sydney, November, 2011. This conference would not have been attended by all those eligible for the study; however the use of a paper based survey was used both to maximize recruitment and to minimise bias that may have occurred due to limited internet access (which can occur in remote or rural areas) and preference for paper based surveys over electronic formats. Paper surveys were collected in a box to preserve anonymity.

All participants (via electronic recruitment and face-to-face) received an invitation to participate and a comprehensive information form outlining participant rights, the length of the survey, and the purpose of the study which was to assess Australasian emergency doctors' knowledge and confidence for dental emergencies. Survey commencement was taken as implied consent and survey completion took approximately 15 minutes.

### 2.5. Primary Outcome

The primary outcome was the proportion of respondents obtaining a pass mark on the knowledge test. A pass mark was defined* a priori* by a panel of emergency physicians and registrars as >50% total score (at least 8/15) and was calculated by summing correct answers on the knowledge test for those participants who answered all questions.

### 2.6. Sample Size

Based on an ACEM membership of 3405 (1392 FACEM, 2013 Trainees) at the time of survey distribution (personal communication, Jane Macaulay, 9/9/11), a sample size of 346 was required in order to estimate the proportion of respondents passing the knowledge test within a 5% margin of error (assuming a 50% response distribution) at a 95% confidence level. This equates to a 50% pass rate for the knowledge portion of the survey. This figure was chosen as it provides the most conservative estimate of sample size required (i.e., maximizing the number required). Further power analyses indicated that a sample size of 346 would be sufficient to detect a difference in two proportions of 15 percentage points (50% versus 65%) with power at 80% and criterion for significance set at 0.05. For multiple regression analyses, we adhered to the rule of thumb for sample size; that is, number of cases = 50 + (8 × number of predictors).

### 2.7. Data Analysis

Quantitative data were analysed using IBM SPSS Statistics 20.0 (Chicago, IL). For each survey summary statistics (%, 95% confidence interval (CI)) were calculated for the entire sample and by the demographic variables. Arithmetic mean (95% CI) was used to summarize the total number of correct knowledge items across that sample. All data reported were adjusted for missing data on an item by item basis.

Representation of membership per state and membership type was calculated by comparing the confidence intervals (CI) of the percentages of all members of ACEM to the percentages of respondents to the survey. If the CI overlapped, we considered that they were adequately represented.

Negatively worded survey items from the confidence section were reverse scored. Exploratory inferential analyses on confidence and attitudinal items were undertaken after collapsing Likert Scales to binary scales (strongly disagree/disagree/neutral versus agree/strongly agree). Fisher's exact test was used for 2 × 2 contingency tables and Pearson's Chi Square (linear by linear association) was used to identify linear trends for ordinal data (4 × 2 contingency tables).

A total score for overall confidence was calculated by summing collapsed items for confidence. Similarly, a total score was calculated for each participant that completed all items in the knowledge test. Each item was identified as being either correct or incorrect (“incorrect/do not know”) prior to summing. To ensure validity of derived total scores, internal consistency of these items was verified using Cronbach's alpha (*r* = 0.805).

Since the primary outcome (proportion of respondents obtaining a pass mark on the knowledge test) was based on a panel determining that a pass mark be defined as 50% or more, further sensitivity analyses were undertaken comparing mean total knowledge score by Fellow (yes/no), received formal training in dental emergencies (yes/no), attended workshops in dental emergencies (yes/no), ED type and number of patients seen per year (0–25, 26–50, 51+). Data were analysed using independent samples *t*-test and ANOVA in situations where there were 3+ categories.

Multiple regression (“enter” method) was then used to identify (demographic) predictors of knowledge score and all predictors of total confidence. For total knowledge, 11 demographic predictors were assessed: FACEM (no/yes); ED type (adult/other); whether formal education in dental emergencies had been received (yes/no); whether the respondent had participated in a conference workshop on dental emergencies (yes/no), and the number of patients seen annually (continuous); access to specialized dental service coded as six dummy variables (none; onsite dentist 24/7; onsite dentist, limited; dentist on call; refer to dental hospital; refer to private dentist). For confidence, these same predictors were included in the model with the addition of total knowledge score.

For multiple regression, variance inflation factor (VIF) was used to indicate the presence or absence of multicollinearity with a criterion of VIF <5 set for retention of variables. Other assumptions (outliers, linearity, homoscedasticity, and independence of residuals) were assessed by inspecting the residuals scatterplot and the normal probability plot of regression standardised residuals. Pearson's correlation was used to explore an association between knowledge score and total confidence score.

For all inferential tests, alpha was set at 0.05 and two tailed tests of significance were used.

## 3. Results

### 3.1. Participation

Four hundred and ten respondents were recruited via the online survey during the study period and 56 via the paper-based survey. Two participants were not registered with ACEM and were therefore excluded resulting in 464 participants commencing the survey from an eligible pool of 3405 ACEM members (13.6%). Of these, 14 completed the demographics section of the survey only and were excluded from all further analyses. There were no significant differences according to demographics in respondents that commenced the survey (*n* = 464) and those that completed all elements of the survey (*n* = 391).

### 3.2. Demographics

Respondents were from a total of 117 hospitals, and two respondents were fulltime locums. The distribution of respondents by region is comparable to the membership of ACEM (Table 1) except for a slight preponderance of Victorian respondents and fewer respondents from Queensland proportional to the ACEM membership. Respondents identified working mainly at EDs that receive both adults and children (adult: 108/449, 24.1%; paediatric only: 13/449, 2.9%; mixed: 328/449, 73.1%). Data for paediatric and mixed hospitals were collapsed for subsequent analyses.

Approximately one fifth of participants (85/447, 19.0%) had not completed the primary exam and almost half the respondents (204/434, 47.0%) had not yet completed the fellowship exam. This was substantially less than the proportion of trainees registered with ACEM at the time of the study (2013/3405, 59.1%). Of respondents who had completed the fellowship, 16.6% (72/434) obtained the fellowship during or prior to 2000; 16.4% (71/434) between 2001 and 2005; and 20.0% (87/434) after 2005.

**Figure 1:**
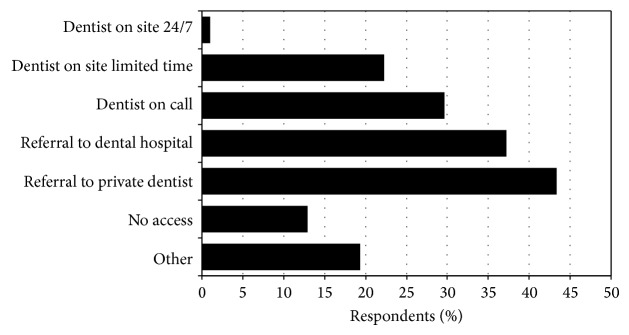
Percentage of respondents according to access to dental services from ED (more than one response accepted).

One hundred and eighty six respondents (41.7%) reported having received some formal training in dental emergencies, while 121/447 (27.1%) indicated participating in either a conference or workshop specifically on dental emergencies. Respondents indicated having limited access to a dentist in ED (Figure 1).

### 3.3. Knowledge of Dental Emergencies

In total, 391 respondents completed all knowledge test items. For those participants, the mean (95% CI) total score out of a possible 15 was 8 (7.8–8.3; range 1–14). A total of 244/391 (62.4%, 95% CI 57.5–67.1) participants completing all items achieved a pass (>50%) in the knowledge test. Knowledge of dental emergencies varied considerably across topics (Table 2). The majority of respondents obtained 6–10 items correctly not adjusted for missing data; 284/417, 68.1%, (95% CI 63.5–72.4) with the remainder fairly evenly distributed in the 1–5 range (68/417, 16.3%, 95% CI 13.1–20.2) and the 11–15 range (65/417, 15.6%, 95% CI 12.4–19.4).

Sensitivity analyses indicated that those that had attended formal education and workshops in dental emergencies, and those that were fellows obtained significantly higher total confidence scores compared to their counterparts. There were no significant differences according to ED type and number of patients seen per year (data not shown).

### 3.4. Confidence in Dental Emergency Related Tasks

**Figure 2:**
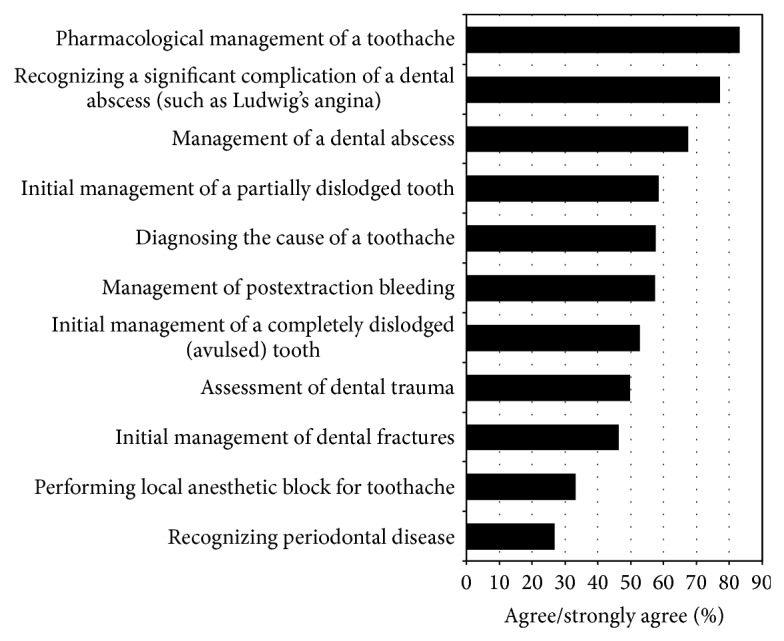
Practitioner confidence in specific topics of dental emergencies.

Confidence for tasks relating to the management of dental emergencies ranged from 26.6% of participants (95% CI 22.7–30.8%, 119/448) reporting confidence in recognition of periodontal disease to 82.9% (95% CI 79.1–86.1, 372/449) for pharmacological management of toothache (Figure 2). Receipt of formal dental education was significantly associated with confidence for all tasks apart from pharmacological management of toothache and managing a dental abscess (Table 3).

**Figure 3:**
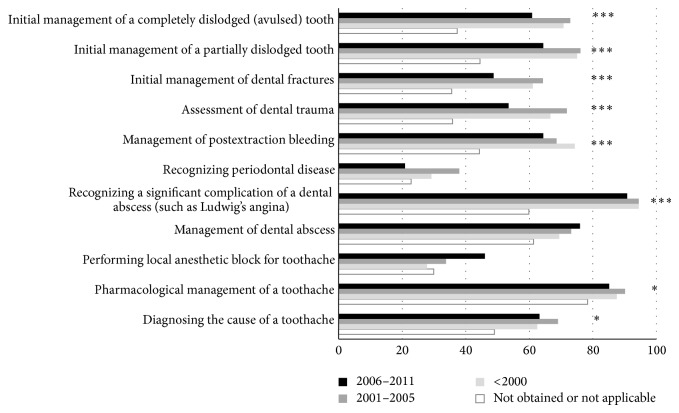
Practitioner confidence (agreed/strongly agreed) in specific skills relating to dental emergencies according to year the Fellowship was obtained. ^*^denotes *P* < 0.05. ^***^denotes *P* < 0.001.

Confidence varied significantly by hospital type for several tasks: performing local anesthetic block for toothache (*P* = 0.026; adult hospitals: 26/108, 24.1%, 95% CI 16.9–33.0; other hospitals: 122/341; 35.8%, 95% CI 30.9–41.0); recognizing significant complications of abscess (*P* = 0.036; adult: 75/108, 69.4%, 95% CI 60.2–77.4; other: 269/339, 79.4%, 95% CI 74.7–83.3); and managing the completely avulsed tooth (*P* = 0.036; adult: 47/108, 43.5%, 95% CI 34.6–52.9; other: 188/340; 55.3%, 95% CI 50.0–60.5). Confidence also varied significantly by recency of graduation as a specialist, with more senior graduates more confident in many areas (Figure 3). Total confidence score was positively correlated with total knowledge score (*r* = 0.488, *P* < 0.001). Significant linear relationships existed between confidence for most tasks explored and year fellowship obtained, with a trend toward a greater level of confidence and earlier attainment of the fellowship (Figure 3).

### 3.5. Predictors of Knowledge and Confidence

A significant model emerged for predictors of knowledge (*F*_(11,348)_ = 6.722, *P* < 0.001; adjusted *R* square = 0.149) and also for predictors of total confidence score (*F*_(12,350)_ = 23.545, *P* < 0.001; Adjusted *R* square = 0.428). For knowledge, the strongest predictor was being a FACEM, whereas the strongest predictor of confidence was receipt of formal education. Other significant predictors of knowledge were having attended a conference workshop in dental emergencies and having received formal training in dental emergencies (Table 4). Other significant predictors for confidence were being a FACEM having attended a conference workshop in dental emergencies, having a dentist on call, total score on the knowledge test, and number of patients seen (Table 4).

### 3.6. Attitudes towards Knowledge and Training in Dental Emergencies

The majority of respondents (393/405, 97.0%, 95% CI 94.8–98.4) agreed or strongly agreed that it was important for emergency trainees to have practical knowledge about dental emergencies. Less than one percent (3/408; 0.7%, 95% CI 0.2–2.2) agreed or strongly agreed that there was too much attention on dental emergencies in the current emergency medicine training program.

There were no significant attitudinal differences according to year of fellowship, receipt of formal education, or participation in workshops or conferences on dental emergencies.

### 3.7. Continuing Professional Development

The preferred format of future continuing professional development (CPD) activities relating to dental emergencies revealed a strong preference for interactive workshops (386/415, 93.0%, 95% CI 90.1–95.1), watching visual presentations (357/415, 86.0%, 95% CI 82.3–89.1), didactic lectures (333/415, 80.2%, 95% CI 76.1–83.8), and case-based conferences (331/415, 79.8%, 95% CI 75.6–83.4). Fewer respondents indicated a desire for CPD obtained through the internet (318/415, 76.6%, 95% CI 72.3–80.5), textbooks (295/415, 71.1%, 95% CI 66.5–75.2), and listening to audio presentations (293/415, 70.6%, 95% CI 66.0–74.8). Other suggested CPD methods included a short rotation at an acute dental clinic or dental hospital ED, bedside teaching with a dentist or maxillofacial surgeon, a course, and on the job teaching.

When asked to indicate the preferred frequency of live education programs (e.g., lectures and workshops) on dental emergencies, the modal responses were annually (200/405; 49.4%, 95% CI 44.5–54.2) and every six months (115/405; 28.4%, 95% CI 24.2–33.0). The most commonly preferred duration of live education programs ranged between half a day (160/404, 39.6%, 95% CI 35.0–44.5) and 2 hours (139/404, 34.4%, 95% CI 29.9–39.2).

## 4. Discussion

Patients with dental problems present to the ED [3, 22] mostly on weekends and outside normal working hours, when dentists might not be available. One third of Australian adults do not visit the dentist because of expenses [1, 23]. An adequate understanding of dental disease and trauma is important for ED physicians to be able to diagnose, treat, and refer patients with dental emergency efficiently [24]. Poor knowledge can result in premature referral, where management is possible by the emergency doctor but is not provided, or late referral in situations when a tooth- or life-threatening problem exists.

To our knowledge, this is the first study to assess the knowledge and confidence of Australasian Emergency Physicians and Trainees' with respect to dental emergencies. We have demonstrated that knowledge and confidence vary considerably and that they are intimately related. Both are enhanced by seniority and experience working in emergency medicine and by participation in teaching programs or educational activities. Clinicians who scored well on the knowledge score were justifiably confident in their ability to manage dental emergencies.

We found knowledge to be better in some areas than others. More than 70% of respondents correctly answered questions relating to common dental conditions such as tooth avulsion, antibiotics treatment, and treatment of dry sockets. Less than half the surveyed doctors however correctly answered questions about dental abscesses, dental fractures, and ulcerative gingivitis. A previous study [10] of 30 new graduates reported that “no physician” showed a high level of knowledge with respect to managing tooth avulsion. This contrasts with more than 80% of all respondents in our study who correctly answered this knowledge question.

While confidence is not an indicator for competence, it may affect the decisiveness of management. In our study, the range of confidence varied from 27% (95% CI 22.7–30.8) in recognition of periodontal disease to 83% (95% CI 79.1–86.1) for pharmacological management of toothache. Almost 70% of FACEMs and 37% of trainees in our study were confident in managing tooth avulsion which conflicts with a recent UK study [4] among 120 ED physicians with only 20% of them confident in managing dental avulsion injuries. Surprisingly, doctors' self-rated confidence was high in managing dental fractures and abscesses despite their lack of knowledge in these areas. They did however recognize their lack of knowledge in recognizing periodontal disease.

Confidence and knowledge were moderately strongly correlated, and knowledge was a significant predictor of confidence suggesting that the two are intimately related. It is possible that being a FACEM may increase confidence generally, but clearly education plays an important part in both knowledge and confidence since being a FACEM, having received formal training in dental emergencies, and having attended a conference workshop in dental emergencies were significant predictors of both.

These findings suggest that specific education in dental emergencies may be beneficial for doctors working in EDs. Virtually all respondents agreed that it was important for emergency trainees to have practical knowledge of dental emergencies. Interactive workshops were the most highly valued educational format, supported by visual presentations, lectures, and case-based conferences. This is unsurprising since interactive workshop which was most preferred format of future CPD in our study, has significant effect on professional practice based on a Cochrane Database Systemic Review [25]. Most members of the ACEM feel that such training should be provided once or twice a year, for a two hour to half day period. The ACEM may consider offering more sessions on dental emergencies at their annual scientific meetings, potentially involving dental practitioners with particular background, and expertise in dental emergencies.

An interesting finding of the recent UK study [7] showed that only 3.9% of ED physicians would choose to be seen and treated by another ED doctor if they had a traumatic dentofacial injury. Seventy-two percent preferred to be treated by a maxillofacial surgeon and 23.5% by a dentist [4]. While this may be a reflection of attitudes towards emergency clinicians dealing with dental injuries, it is also likely that survey respondents are aware of the “system” and the likely referral to specific dental or faciomaxiallary clinicians for definitive care.

The data from this study reinforce anecdotal evidence that access to dental services in emergency situations is very limited. This lack of access makes it even more important that emergency clinicians have adequate knowledge to manage dental emergencies. Referral off-site to other providers may delay treatment, increase morbidity, and inconvenience patients. Emergency clinicians should be able to manage most of the common emergencies in the ED.

Many dental emergencies rely on the clinician's ability to provide adequate pain relief, including local anesthetic blockade. Although this skill is a procedure for which a high level of competence is expected among ACEM members, only one third of respondents reported that they could confidently use local anesthetics in toothache. Regional anaesthesia techniques, including dental anaesthesia, were rated as one of the most requested education procedures by ACEM fellows in a national learning need analysis [26]. This analysis suggested that high quality and well explained videos and regular workshops could help members improve this core competency, as suggested by respondents to our survey. Our finding that only 17% of members without formal education could perform local anesthetics compared with 46% with some kind of formal education, supports the value of such educational activities.

## 5. Limitations

This study is not without limitation. The overall response rate was low which is not uncommon for survey research and may be lower among this cohort due to the possibility of eligible participants being asked to complete multiple survey-based research projects via the ACEM (Dr Andrew Gosbell, personal communication), thereby resulting in survey fatigue. The distribution of respondents by region was however comparable to the eligible study population, except for a slight preponderance of Victorian respondents and fewer respondents from Queensland. This suggests the sample was reasonably representative of ACEM members. Further demographic information about the total ACEM population was not available to the authors and therefore restricted further assessment of representativeness. The use of ACEM as the organization distributing the email to potential participant was an ethical requirement for the study due to privacy laws but is likely to have resulted in the use of correct email addresses as ACEM is the professional body that manages specialist status in Australasia for emergency physicians.

We attempted to minimise selection bias by recruiting participants through the two methods described: an online survey and by direct distribution of the survey to eligible registrants at the ACEM Annual Scientific Meeting. The direct recruitment approach was used in order to enroll eligible doctors whose participation in the study through completion of the online questionnaire was unlikely due to internet access barriers or a bias against online survey completion. We cannot exclude the possibility of responder bias; those more interested in dental emergencies may have been more likely to respond to the survey. If such effect were present this may have produced an overestimate the actual knowledge and confidence of ACEM members.

We cannot exclude the possibility of responder bias; those more interested, competent, or confident in dental emergencies may have been more likely to respond to the survey, and those less comfortable with their knowledge may have been less inclined to respond. The possible survey fatigue among the target group may have biased participation to those who are more engaged and possibly therefore more likely to have been involved in educational and other college activities; this might also have resulted in higher test scores.

We attempted to minimise measurement bias by establishing face validity and content validity of the tool, given the absence of a previously validated tool on dental emergencies. Additionally, we undertook further validation of the internal consistency of the knowledge component and confidence component of the tool using Cronbach's alpha. Although the multiple choice questions that comprised the knowledge assessment in this survey were reviewed by several ED consultants and registrars, it was not an exhaustive examination of dental emergencies, assessing just a few key presentations and therefore may not extrapolate well to other dental emergencies not included in the survey. Further, although the pass mark of 50% was set* a priori* by a panel of emergency physicians, this cutoff may be deemed by some as somewhat arbitrary; however the findings of sensitivity analyses were in line with the expectation that seniority and exposure to education and training would result in higher total scores.

We did not instruct participants to avoid consulting educational materials to improve performance. The anonymous nature of the survey, however, may have minimised any such Hawthorne Effect.

## 6. Conclusions

The knowledge and confidence of Australasian ED doctors with respect to dental emergencies is varied, being good in some areas but in need of improvement in others. Seniority in the ED is associated with both confidence and knowledge. However, formal interactive education is also associated with confidence and knowledge, and importantly, is desired by the majority of specialist and trainee emergency physicians in Australasia. The use of designated training workshops on dental emergencies is therefore warranted.

## Figures and Tables

**Table 1:** Distribution of respondents by region [number (percentage; 95% CI)].

	Survey respondents	ACEM members
VIC	143 (31.8; 27.6–36.2)	772 (22.7; 21.3–24.1)
NSW	106 (23.6; 19.9–27.7)	820 (24.1; 22.7–25.6)
QLD	65 (14.4; 11.5–18.0)	705 (20.7; 19.4–22.1)
WA	44 (9.8; 7.3–12.9)	321 (9.4; 8.5–10.5)
NZ	37 (8.2; 6.0–11.2)	337 (9.9; 8.9–11.0)
SA	25 (5.6; 3.8–8.1)	214 (6.3; 5.5–7.2)
TAS	12 (2.7; 1.5–4.7)	78 (2.3; 1.8–2.9)
NT	7 (1.6; 0.7–3.2)	44 (1.3; 1.0–1.7)
ACT	5 (1.1; 0.4–2.7)	51 (1.5; 1.1–2.0)
Overseas	6 (1.3; 0.5–3.0)	63 (1.9; 1.5–2.4)
Total	450 (100.0)^*^	**3405 (100.0)**

^*^14 respondents did not specify their region.

**Table 2:** Percentage (95% CI) of participants answering knowledge item correctly.

Item	% Correct (95% CI); numerator/denominator
Acute necrotizing ulcerative gingivitis knowledge	**7.0** (4.9–9.9); 29/413
Tooth eruption dates	**18.1** (14.7–22.1); 75/415
Periodontal abscess knowledge	**25.3** (21.3–29.7); 104/411
Dental fracture knowledge	**38.6** (34.1–43.4); 160/414
Dry socket management	**52.9** (48.1–57.7); 217/410
Ludwig's angina knowledge	**59.7** (54.9–64.3); 246/412
Reimplantation of a deciduous avulsed tooth?	**59.7** (54.9–64.3); 247/414
Full dentition description	**60.2** (55.4–64.8); 251/417
Dry socket knowledge	**70.3** (65.7–74.5); 291/414
Aphthous stomatitis knowledge	**70.5** (66.0–74.7); 292/414
Causes of oral pain	**70.9** (66.4–75.8); 295/416
Antibiotics for dental pain	**75.2** (70.9–79.2); 313/416
Tooth avulsion knowledge	**81.6** (77.6–85.1); 338/414
Naming of teeth	**83.9** (80.1–87.2); 350/417
Tooth avulsion advice	**89.4** (86.1–92.1 ); 372/416

**Table 3:** Participants who agreed/strongly agreed to being confident in specific skills according to receipt of formal education and participation in workshops and conferences.

Item/skill	Received formal education			Participated in a conference or workshop on dental emergencies		
	Yes	No	*P* value^†^	Yes	No	*P* value^†^
Diagnosing the cause of a toothache	134/186 72.0% 65.2–78.0	123/259 47.5% 41.5–53.6	**<0.001**	84/121 69.4% 60.7–77.0	174/325 53.5% 48.1–58.9	**0.003**

Pharmacological management of a toothache	175/185 94.6% 90.2–97.2	193/280 74.2% 63.3–47.9	**<0.001**	106/121 87.6% 80.4–92.5	264/325 81.2% 76.6–85.1	0.121

Performing local anesthetic block for toothache?^*^	81/149 54.4% 46.4–62.2	105/297 35.4% 30.1–41.0	**<0.001**	68/148 45.9% 38.1–54.0	53/299 17.7% 13.8–22.5	**<0.001**

Management of a dental abscess?	141/186 75.8% 69.2–81.4	159/280 61.2% 50.9–62.5	**0.001**	87/121 71.9% 63.3–79.2	215/326 66.0% 60.6–70.9	0.257

Recognizing a significant complication of a dental abscess (such as Ludwig's angina)	158/185 85.4% 79.6–89.8	185/259 71.4% 65.6–76.6	**0.001**	106/121 87.6% 80.4–92.5	238/324 73.5% 68.4–78.0	**0.001**

Recognizing periodontal disease?^*^	56/119 47.9% 38.3–56.0	129/325 39.7% 34.5–45.1	0.192	50/118 42.4% 33.8–51.4	70/257 27.2% 22.2–33.0	**<0.001**

Management of postextraction bleeding?	128/186 68.8% 61.8–75.1	122/254 48.0% 42.0–54.2	**<0.001**	83/119 69.7% 61.0–77.3	169/323 52.3% 46.9–57.7	**0.001**

Assessment of dental trauma?^*^	129/220 58.6% 52.0–65.0	60/224 26.8% 21.4–33.0	**<0.001**	88/222 39.6% 33.4–46.2	33/223 14.8% 10.7–20.1	**<0.001**

Initial management of dental fractures?	118/185 63.8% 56.6–70.4	87/257 33.9% 28.3–39.8	**<0.001**	89/119 74.8% 66.3–81.8	116/324 35.8% 30.8–41.2	**<0.001**

Initial management of a partially dislodged tooth?	143/186 76.9% 70.3–82.4	115/256 44.9% 39.0–51.1	**<0.001**	99/121 81.8% 73.9–87.7	160/322 49.7% 44.3–55.1	**<0.001**

Initial management of a completely dislodged (avulsed) tooth?	123/234 52.6% 46.2–59.1	62/211 29.4% 23.6–35.9	**<0.001**	88/235 37.4% 31.5–43.8	33/211 15.6% 11.3–21.2	**<0.001**

^†^Inferential analyses conducted using Fisher's exact test.

^*^Negatively worded items reverse coded before collapsing.

**Table 4:** Predictors of knowledge and confidence in dental emergencies.

	Knowledge			Confidence		
	*P*	Beta	95% CI	*P*	Beta	95% CI
(Constant)	<0.001	3.223	1.713–4.733	<0.001	−3.990	−5.550–2.431
ED type (adult/other)	0.609	0.153	−0.433–0.738	0.310	0.299	−0.280–0.878
Received formal education in dental emergencies	**0.023**	0.592	−0.082–1.102	**<0.001**	1.827	1.320–2.333
Participated in conference or workshop specifically about dental emergencies	**0.002**	0.926	0.337–1.516	**<0.001**	1.064	0.486–1.643
Number of dental patients seen annually	0.512	−0.001	−0.004–0.002	**0.026**	0.003	0.000–0.006
Fellow (no/yes)	**<0.001**	1.438	0.927–1.949	**<0.001**	1.114	0.590–1.638
No access^*^	0.417	0.357	−0.508–1.222	0.830	−0.092	−0.932–0.749
Dentist on site 24/7^*^	0.945	0.117	−3.223–3.457	0.342	1.314	−1.405–4.033
Dentist on site (limited)^*^	0.209	0.382	−0.214–0.978	0.100	0.497	−0.096–1.089
Dentist on call^*^	0.841	−0.058	−0.631–0.514	**0.004**	0.824	0.261–1.386
Refer to dental hospital^*^	0.274	0.308	−0.245–0.862	0.787	0.076	−0.477–0.629
Refer to private dentist^*^	0.228	0.328	−0.206–0.863	0.843	0.054	−0.479–0.586
Total knowledge score	N/A	N/A	N/A	**<0.001**	0.421	0.315–0.527

^*^Option when asked “What access does your ED have to specialist dental services?”

## Appendix

The appendix contains the questionnaire items presented to respondents. It includes four parts. Part one requests demographic information. Part two requests respondents to rate their confidence in dental emergencies. Part three requests respondents to complete a multiple choice knowledge test of dental emergencies. Part four requests respondents to provide suggestions for further training in dental emergencies.

*Part One (demographics)*

1. In which region do you usually work?
  QLD
  NSW
  VIC
  ACT
  SA
  WA
  NT
  TAS
  New Zealand
2. Which year did you pass the primary exam?
  …
3. Which year did you pass the fellowship exam?
  …
4. In which hospital do you primarily work (The hospital will be de-identified prior to publication)?
5. The ED in which you work is:
  Adult ED
  Paediatric ED
  Mixed ED
6. Have you had any formal education about dental emergencies during your emergency training?
  Yes
  no
7. Have you ever participated in any conference or workshop specifically about dental emergencies?
  Yes
  no
8. How many patients do you see approximately each year with a tooth-related complaint (pain, abscess, trauma, etc)?
9. What access does your ED have to specialist dental services? (you can choose more than one option)
  No access
  Dentist on site (24/7)
  Dentist on site (limited time)
  Dentist on call
  referral to dental hospital
  referral to private dentist
  Other-please specify…

*Part Two (your confidence in dental emergencies)*. Please rate your agreement to the following statements about your confidence in dental emergencies:

1. I am confident in diagnosing the cause of a toothache?
  □ Strongly Disagree
  □ Disagree
  □ Neutral
  □ Agree
  □ Strongly Agree
2. I am confident in the pharmacological management of a toothache?
  □ Strongly Disagree
  □ Disagree
  □ Neutral
  □ Agree
  □ Strongly Agree
3. I* am not* confident in performing local anesthetic block for toothache?
  □ Strongly Disagree
  □ Disagree
  □ Neutral
  □ Agree
  □ Strongly Agree
4. I am confident in the management of a dental abscess?
  □ Strongly Disagree
  □ Disagree
  □ Neutral
  □ Agree
  □ Strongly Agree
5. I am confident in recognizing a significant complication of a dental abscess (such as Ludwig's angina)
  □ Strongly Disagree
  □ Disagree
  □ Neutral
  □ Agree
  □ Strongly Agree
6. I* am not* confident in recognizing periodontal disease?
  □ Strongly Disagree
  □ Disagree
  □ Neutral
  □ Agree
  □ Strongly Agree
7. I am confident in the management of post-extraction bleeding?
  □ Strongly Disagree
  □ Disagree
  □ Neutral
  □ Agree
  □ Strongly Agree
8. I* am not* confident in the assessment of dental trauma?
  □ Strongly Disagree
  □ Disagree
  □ Neutral
  □ Agree
  □ Strongly Agree
9. I am confident in the initial management of dental fractures?
  □ Strongly Disagree
  □ Disagree
  □ Neutral
  □ Agree
  □ Strongly Agree
10. I am confident in the initial management of a partially dislodged tooth?
  □ Strongly Disagree
  □ Disagree
  □ Neutral
  □ Agree
  □ Strongly Agree
11. I* am not* confident in the initial management of a completely dislodged (avulsed) tooth?
  □ Strongly Disagree
  □ Disagree
  □ Neutral
  □ Agree
  □ Strongly Agree

*Part Three (knowledge test of dental emergencies)*. In the following questions, choose the single best answer. If you are not sure about the correct answer, please choose the last option (e)

1. Full dentition in an adult and in a child consists of:
  - 28 permanent teeth, 20 deciduous teeth
  - 32 permanent teeth, 20 deciduous teeth
  - 28 permanent teeth, 16 deciduous teeth
  - 32 permanent teeth, 16 deciduous teeth
  - I am not sure
2. What is the name of the left upper fourth tooth from the midline in the adult dentition?
  - Incisor
  - Canine
  - Premolar
  - Molar
  - I am not sure
3. Regarding eruption date of teeth, which one of the following statements is* incorrect*?
  - At about 6 years of age, the first permanent teeth begin to erupt
  - Between approximately 6 and 12 years age, children have a mix of permanent and deciduous teeth
  - The first permanent molars (upper and lower) and lower incisors are the first permanent teeth to erupt
  - By the age of 12 most children have all their permanent teeth
  - I am not sure
4. Which one of the following is incorrect about Aphthous Stomatitis?
  - The lesions are tender
  - The condition is self limiting
  - An antibiotic is usually given to prevent secondary infection
  - It may occur following oral trauma
  - I am not sure
5. What is the most common source of oral pain?
  - Maxillary sinusitis
  - Dental caries
  - Cracked tooth
  - Root canal pain
  - I am not sure
6. When would you consider re-implantation of a primary (deciduous) avulsed tooth?
  - Always
  - Never
  - Depends on the age of child
  - Depends on the child's cooperation
  - I am not sure
7. Which one of the following is* incorrect* regarding dental fracture?
  - In Ellis class III fracture the pulp is involved
  - Ellis class II fracture is more serious in children because of less dentin to protect the pulp after exposure to the oral cavity
  - The most common dental fracture involves pulp exposure
  - pulp exposure is a true dental emergency and a dentist should be notified immediately
  - I am not sure
8. Which of the following statements is incorrect regarding tooth avulsion?
  - Foreign bodies on the root may be gently removed with forceps
  - Remove any blood from the tooth with alcohols/anti-septic irrigation
  - The tooth must not be allowed to dry out, it may be stored in milk for a short period
  - A permanent tooth should be replaced into its socket as soon as possible, ideally within 2 hours
  - I am not sure
9. After an accidental injury, a 30 year old patient calls the ED for advice about an avulsed front tooth. The first advice that you give to the patient is:
  - Wrap the tooth in a tissue and see a dentist
  - Scrub and clean the tooth and put it back in the socket
  - Wash the tooth in the running warm water, then clean the root of the tooth with gauze, put it in the socket, see a dentist as soon as possible
  - Wash it in the running cold water, do not touch the root of the tooth, put it in the socket and if not possible, store in milk, see a dentist as soon as possible
  - I am not sure
10. You generally give an antibiotic to a patient with dental pain in all following situations* except*?
  - Pain not responding to analgesia
  - Systemic symptom (e.g., fever)
  - Regional lymphadenopathy
  - Facial swelling
  - I am not sure
11. A patient presents to ED 2 days after a routine tooth extraction, complaining of pain in the same site where the tooth was extracted. The pain is much worse than the original toothache. On examination the patient is afebrile and there is food debris in the socket. What is the most likely diagnosis?
  - Osteomyelitis
  - Alveolar osteitis (dry socket)
  - Retained root
  - Incorrect extraction
  - I am not sure
12. Regarding “acute necrotizing ulcerative gingivitis”, which one of the following statements is* incorrect*?
  - The diagnostic triad includes pain, ulcerated interdental papillae, and gingival bleeding
  - The most important predisposing factor is human immunodeficiency virus (HIV)
  - Unusual emotional stress is one of the contributing factors
  - The peak age of incidence is early 40's
  - I am not sure
13. The following statements are correct about “periodontal abscess”* except*:
  - The tooth is usually mobile
  - It usually causes extra-oral swelling
  - The tooth is tender to percussion
  - There is swelling of adjacent gingiva
  - I am not sure
14. How do you manage a patient with alveolar osteitis (dry socket)?
  - Give analgesia and send him to see the same dentist who extracted the tooth
  - Start antibiotic and analgesia and send him to his dentist
  - Check the socket for any discharge or infection and then irrigate the socket, pack the socket with sterile gauze soaked in local anesthetic, then send him to see a dentist
  - Give chlorhexidine mouthwash and prescribe antibiotic and analgesia and send him to see a dentist
  - I am not sure
15. Which one of the following statements is incorrect regarding Ludwig's angina?
  - The most serious immediate sequela is airway obstruction
  - Hemolytic streptococcus is most commonly responsible for the infection
  - A site of discrete fluctuance can often be found
  - It is bilateral swelling involving the submandibular, submental and sublingual spaces
  - I am not sure

*Part Four (suggestions about further training in dental emergencies)*. Please enter your agreement/disagreement (where applicable) with the following statements:

1. It is important for emergency trainees to have practical knowledge about dental emergencies:
  □ Strongly Disagree
  □ Disagree
  □ Neutral
  □ Agree
  □ Strongly Agree
2. There is too much attention on dental emergencies in the current emergency medicine training program:
  □ Strongly Disagree
  □ Disagree
  □ Neutral
  □ Agree
  □ Strongly Agree
3. Which of the following types of education about dental emergencies would you undertake [please choose as many as you want and prioritise them with number (1-2-3…)]:
  - didactic lectures
  - interactive workshops
  - case-based conferences
  - textbook
  - internet
  - listening to audio presentations
  - watching visual presentations
  - other
4. How frequently should there be live educational program (lectures, workshops …) about dental emergencies available for ED trainees?
  Never
  every 6 months
  every year
  every 18 months
  every 2 years
  every 5 years
5. What is the optimal duration for a live educational program (lectures, workshops…) on dental emergencies?
  1 hour
  2 hours
  Half day
  Full day
  Multi-day
  Other-(please specify)…
